# Does the Duration of FLOT Infusion Change the Outcome of Perioperative Treatment for Gastric Cancer? Comparing 24- and 48-h Infusions

**DOI:** 10.3390/medicina62050987

**Published:** 2026-05-19

**Authors:** Hacer Demir, Canan Yıldız, Yusuf İlhan, Murat Araz, Ali Fuat Gürbüz, Semiha Urvay, Muslih Urun, Berrak Mermit Ercek, Onur Yazdan Balçık, Beyza Ünlü, Sena Ece Davarcı, Ramazan Cosar, Meltem Baykara, Ismail Beypinar

**Affiliations:** 1 Department of Medical Oncology, Afyonkarahisar Health Sciences University, Afyon 03030, Turkey; dr-beyza@hotmail.com (B.Ü.); beyaaz3@hotmail.com (S.E.D.); ramazancosar@gmail.com (R.C.); meltembaykara@yahoo.com (M.B.); 2 Department of Medical Oncology, Antalya City Hospital, Antalya 07080, Turkey; yusufilhan_1988@hotmail.com; 3 Department of Medical Oncology, Necmettin Erbakan University, Konya 42080, Turkey; zaratarum@yahoo.com (M.A.); dr.alifuatg@gmail.com (A.F.G.); 4 Department of Medical Oncology, Kayseri Acıbadem Hospital, Kayseri 38030, Turkey; s.elmaci@yahoo.com; 5 Department of Medical Oncology, Van Yüzüncü Yıl University, Van 65080, Turkey; muslihurun@gmail.com (M.U.); drmermitberrak@gmail.com (B.M.E.); 6 Department of Medical Oncology, Alaaddin Keykubat University, Antalya 07425, Turkey; onuryazdanbalcik@gmail.com (O.Y.B.); ibeypinar@hotmail.com (I.B.)

**Keywords:** gastric cancer, FLOT, infusion duration, toxicity, survival, propensity score matching

## Abstract

*Background and Objectives:* FLOT is a highly effective first-line treatment for metastatic gastric cancer and offers a favorable safety profile. Clinical studies investigating the FLOT regimen have reported varying outcomes depending on the infusion duration and have highlighted possible differences in complication rates and the efficacy of neoadjuvant therapy. The choice between 24 h or 48 h infusion durations for fluorouracil can be influenced by several factors, such as the patient’s overall health status, their tolerance to treatment, and the specific treatment protocol determined by the medical team. In this study, we aimed to evaluate the effects of different infusion durations (24 and 48 h) on clinical response, toxicity, and survival in patients with gastric and gastroesophageal junction (GEJ) adenocarcinoma. *Materials and Methods:* This retrospective multicenter study included 113 patients with gastric or gastroesophageal junction adenocarcinoma who received neoadjuvant FLOT chemotherapy (24 h infusion: *n* = 28; 48 h infusion: *n* = 85). Propensity score matching (PSM) was performed to balance baseline characteristics, yielding a matched cohort of 90 patients. The primary endpoints were the pathologic complete response (pCR) and toxicity. Secondary endpoints included disease-free survival (DFS) and overall survival (OS). *Results:* Significant baseline imbalances existed (cT stage *p* < 0.001). After PSM, the balance improved (cT stage *p* = 0.009). In the matched cohort, pCR 11.1% (24 h) vs. 12.1% (48 h), *p* > 0.99. The median DFS was 27.4 mo (24 h) vs. NR (48 h), *p* = 0.847. The median OS was 32.8 mo in both, *p* = 0.797. Multivariate analysis (baseline variables) indicates that infusion duration is not prognostic (DFS HR = 0.77, *p* = 0.453; OS HR = 0.72, *p* = 0.328). Power was ~10% for a 1% pCR difference. *Conclusions:* The 24 h infusion protocol was associated with similar outcomes to the 48 h protocol after PSM adjustment. However, residual confounding persists (cT stage *p* = 0.009 despite PSM), and the combination of this study’s retrospective design and severe underpowering (~10%) precludes definitive conclusions. As a result, the findings are hypothesis-generating.

## 1. Introduction

Gastric cancer (GC), specifically gastroesophageal junction (GEJ) adenocarcinoma, is a major global health burden, with approximately one million new cases and 769,000 deaths estimated worldwide in 2020, making it the fifth most common malignancy and the fourth leading cause of cancer-related mortality [1]. Despite advances in multimodal treatment strategies, the prognosis remains poor for locally advanced disease, with five-year survival rates ranging from 20% to 40% after curative-intent resections [2,3,4]. The introduction of perioperative chemotherapy has significantly improved outcomes in resectable GC and GEJ adenocarcinoma. The landmark FLOT4-AIO (Arbeitsgemeinschaft Internistische Onkologie; fluorouracil, leucovorin, oxaliplatin, and docetaxel) trial demonstrated that perioperative FLOT chemotherapy—comprising docetaxel, oxaliplatin, leucovorin, and 5-fluorouracil (5-FU)—conferred superior overall survival (OS) and pathological complete response (pCR) rates compared to the previously standard ECF (epirubicin, cisplatin, and fluorouracil)/ECX (epirubicin, cisplatin, and capecitabine) regimen in patients with locally advanced, resectable gastric or GEJ adenocarcinoma [5]. This landmark trial established FLOT as the preferred perioperative standard of care [4]. More recently, in the phase 3 MATTERHORN trial, the addition of perioperative durvalumab (an anti-programmed death ligand 1 [PD-L1] immune checkpoint inhibitor) to FLOT has demonstrated a significant improvement in event-free survival compared to FLOT alone, representing a further advancement in treatment [6]. Within the FLOT regimen, 5-FU is administered as a continuous intravenous infusion; however, the optimal infusion duration has not been definitively established [7,8]. In the original FLOT4 trial, 5-FU was delivered via a 24 h infusion, yet many institutions have adopted 46–48 h home-based pump protocols derived from the pharmacokinetic principles established in colorectal cancer chemotherapy [9,10,11]. The 48 h infusion schedule offers potential pharmacodynamic advantages through more sustained plasma drug exposure and may reduce peak-concentration-related toxicities. Clinical studies investigating the FLOT regimen have reported varying results depending on the infusion duration, highlighting potential differences in complication rates, tolerability, and the effectiveness of neoadjuvant therapy [12,13,14]. Research indicates that both the timing of surgery after chemotherapy and the infusion method chosen may affect the frequency of perioperative complications, as well as overall treatment effectiveness [15]. These findings underscore the need for a careful evaluation of infusion strategies to optimize patient care and improve surgical outcomes. Despite the widespread clinical adoption of both infusion durations, prospective comparative data remains scarce and real-world evidence is limited. This study was, therefore, designed to examine how different 5-FU infusion durations—specifically 24 h vs. 48 h schedules—within the perioperative FLOT regimen impact treatment effectiveness, pathological responses, patient tolerance, and survival outcomes in patients with gastric and GEJ adenocarcinoma.

## 2. Methods

This retrospective multicenter study compared 24 h vs. 48 h continuous 5-FU infusion schedules within the perioperative FLOT regimen in patients with gastric or GEJ adenocarcinoma treated across six institutions in Turkey. Given the inherent potential for baseline imbalances in observational, non-randomized studies, propensity score matching (PSM) was employed to minimize confounding and improve the comparability of the two groups.

The inclusion criteria were as follows: pathologically confirmed diagnosis of adenocarcinoma; tumors localized in the stomach or GEJ; administration of the FLOT chemotherapy protocol for neoadjuvant purposes; completion of at least two treatment cycles with response evaluation; and availability of progression or survival data during the follow-up period. Suitable patients had clinically resectable, locally advanced disease (cT2-4 and/or cN+; cT, clinical tumor stage; cN, clinical nodal stage), based on endoscopic ultrasound and computed tomography imaging.

Exclusion criteria included the presence of histological neuroendocrine or squamous cell tumors, a history of another simultaneous malignancy, cases with incomplete treatment data, and loss to follow-up.

### 2.1. Treatment Protocols

Patients were divided into two groups based on the duration of the infusion administered:

24 h infusion group (*n* = 28);

48 h infusion group (*n* = 85).

The FLOT regimen consisted of 50 mg/m^2^ of docetaxel (day 1), 85 mg/m^2^ of oxaliplatin (day 1), 200 mg/m^2^ of leucovorin (day 1), and 2600 mg/m^2^ of 5-fluorouracil delivered as a continuous infusion over either 24 h or 48 h and repeated every 14 days. The duration of infusion was determined according to institutional protocols at each participating center. The patients received four cycles of treatment before surgery and another four cycles postoperatively. Doses were modified according to standard toxicity management guidelines. Dose reductions or delays due to toxicity were applied as required and were documented.

Patients can receive treatment in different settings, depending on the infusion method. In this study, those with a central line, peripherally inserted central catheter (PICC) line, or implantable port generally received 5-FU via a portable pump, allowing outpatient (home) therapy after the initial administration. Alternatively, if treated via cannula, patients are usually required to remain hospitalized for the duration of the infusion. The total infusion time for day 1 could extend to approximately four hours at the infusion center, with the 5-FUU infusion continuing on subsequent days [16].

### 2.2. Study Outcomes and Evaluation

A clinical response evaluation was performed using contrast-enhanced thoracoabdominal CT images captured before and after the treatment according to RECIST (Response Evaluation Criteria in Solid Tumors) 1.1. The response categories included complete response (CR), partial response (PR), stable disease (SD), and progressive disease (PD).

The primary endpoint was pathological complete response (pCR), defined as the absence of viable tumor cells in the resected primary tumor and regional lymph nodes (ypT0 ypN0). Secondary endpoints included the ypT0 rate, ypN0 rate, radiological objective response rate (ORR, defined as complete response plus partial response according to RECIST 1.1), disease-free survival (DFS), overall survival (OS), and toxicity profile. Pathological responses were evaluated according to the eighth edition of the American Joint Committee on Cancer (AJCC) Staging System. Toxicity was graded using version 5.0 of the National Cancer Institute Common Terminology Criteria for Adverse Events (NCI-CTCAE).

### 2.3. Follow-Up and Outcome Measures

Patients were followed up clinically and radiologically at three-month intervals beginning at the end of the treatment.

Disease-free survival (DFS) was defined as the time from surgery to recurrence or death.

Overall survival (OS) was defined as the time from the start of the treatment to death from any cause.

Patients who were alive during the follow-up period were considered alive at the time of analysis.

### 2.4. Statistical Analysis

A post hoc power analysis was conducted for primary endpoints.

Multivariate analysis: Cox regression used baseline variables only (age, gender, cT, cN, infusion duration) to avoid overadjustment related to post-treatment mediators (ypT, ypN).

Propensity score matching: To address baseline imbalances, PSM was performed using logistic regression (covariates: age, gender, cT, cN, histology, location, HER2). One-to-two nearest neighbor matching yielded a matched cohort of 90 patients (30; 24 h, 60; 48 h). Balance was assessed using standardized mean differences (SMD < 0.1 = good).

Baseline characteristics were compared using the chi-square test or Fisher’s exact test for categorical variables and Student’s *t*-test or Mann–Whitney U test for continuous variables. Survival curves were generated using the Kaplan–Meier method and compared using the log-rank test. To identify independent prognostic factors for DFS and OS, multivariate Cox regression analysis was performed, including infusion duration, age, sex, clinical T and N stages, pCR status, and pathological T and N stages. Hazard ratios (HRs) were calculated using 95% confidence intervals (CIs). All statistical tests were two-sided, with *p* < 0.05 considered statistically significant. Statistical analyses were performed using the lifelines and SciPy packages in Python 3.9.

### 2.5. Ethical Approval

This study was conducted after obtaining approval from the ethics committee of Afyonkarahisar Health Sciences University and in accordance with the principles of the Declaration of Helsinki. All data were anonymized to ensure patient confidentiality.

## 3. Results

Patient characteristics: A total of 113 patients were included in this analysis: 28 (24.8%) received a 24 h 5-FU infusion, and 85 (75.2%) received a 48 h infusion. The basic characteristics of the patients and tumors are summarized in Table 1. The median age was 61.7 years (range 23–83) and 59.8 years in the 24 h and 48 h groups (*p* = 0.393), respectively. The proportion of males was higher in both groups (78.6% vs. 62.4%, *p* = 0.166), and the majority of tumors were adenocarcinomas (89.3% vs. 88.2%). Importantly, the clinical T stage was significantly higher in the 24 h group (median cT3-4 vs. cT2-3, *p* < 0.001), which constitutes a potential confounding factor. Dose delays were also more frequent in the 24 h group (35.7% vs. 12.9%, *p* = 0.012). Human epidermal growth factor receptor 2 (HER2) and microsatellite instability (MSI) statuses were balanced between the groups.

### 3.1. Pathological Response

In the matched cohort, pCR was achieved in 3/27 patients (11.1%) in the 24 h group and 7/58 patients (12.1%) in the 48 h group (*p* > 0.99). In the original (unmatched) cohort, the pCR rate was 10.7% (3/28) in the 24 h group and 12.9% (11/85) in the 48 h group (*p* > 0.99), demonstrating the robustness of the findings (Table 2).

**Table 2:** Pathologic response rates.

Outcome	24 h (*n* = 28)	48 h (*n* = 85)	*p*
Pathologic complete response (pCR), *n* (%)	3 (10.7)	11 (12.9)	1
ypT0, *n* (%)	3 (10.7)	12 (14.1)	1
ypN0, *n* (%)	9 (32.1)	34 (40.0)	0.648
Radiologic objective response (CR + PR), *n* (%)	14 (50.0)	42 (49.4)	1

pCR, pathologic complete response (ypT0 ypN0); ypT0, no residual primary tumor; ypN0, no residual nodal disease; CR, complete response; PR, partial response. All comparisons *p* > 0.05.

**Figure 1:**
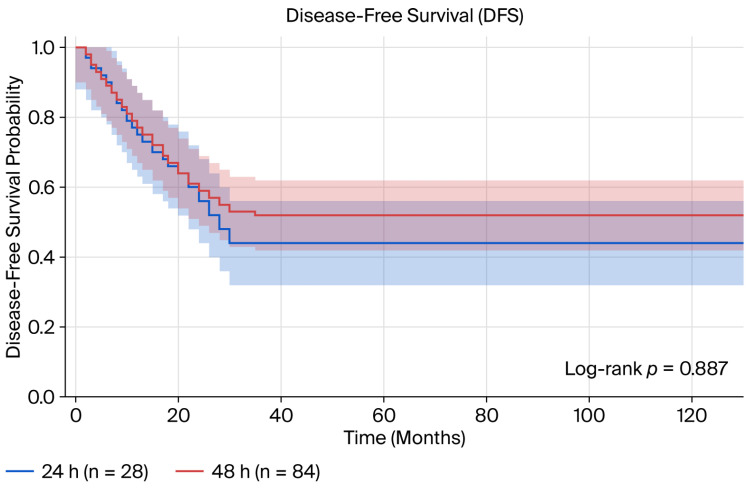
Kaplan–Meier curve for disease-free survival comparing 24 h vs. 48 h 5-FU infusion durations.

**Figure 2:**
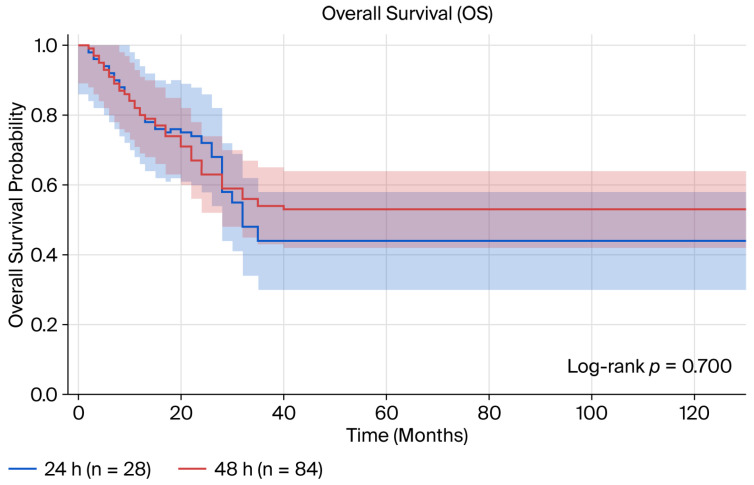
Kaplan–Meier curve for overall survival comparing 24 h vs. 48 h 5-FU infusion durations.

Survival outcomes during a mean follow-up period of 24.3 months: No significant difference was observed in survival outcomes between the infusion duration groups (Table 3, Figure 1 and Figure 2). For disease-free survival, the mean DFS was 27.4 months in the 24 h group, while it had not yet been reached in the 48 h group (log-rank *p* = 0.887). The 1-year and 2-year DFS rates were, respectively, 74.4% and 65.1% in the 24 h group and 72.3% and 61.6% in the 48 h group. The overall survival analysis revealed that the mean OS in the 24 h group was 32.8 months, whereas it has not yet been reached in the 48 h group (log-rank *p* = 0.700). The 1-year and 2-year overall survival rates were 88.4% and 75.7%, respectively, while in the previous period, these rates were 85.5% and 65.7%.

### 3.2. Multivariate Analysis

**Multivariate analysis note:** This table reports the REVISED analysis results using baseline variables only. Post-treatment variables (ypT, ypN) were removed to avoid an overadjustment bias. The 5-FU infusion duration was not an independent prognostic factor after adjusting for baseline tumor characteristics.

**Figure 3:**
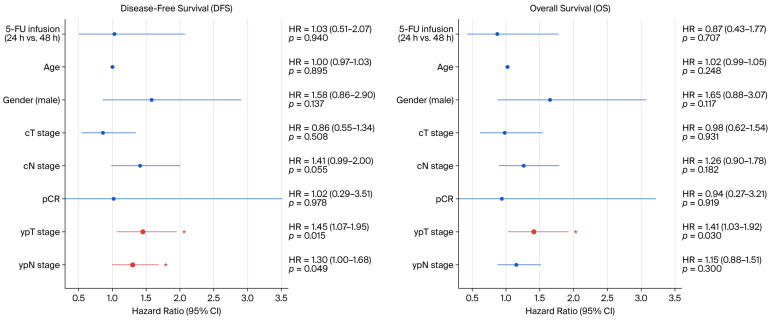
Forest plot of multivariate Cox regression analysis for disease-free survival (**left**) and overall survival (**right**). Asterisks indicate statistical significance (*p* < 0.05).

The multivariate Cox regression analysis (Table 4, Figure 3) identified the pathological T stage as an independent prognostic factor for both DFS (hazard ratio [HR] =1.45, 95% CI 1.07–1.95, *p* = 0.015) and OS (HR = 1.41, 95% CI 1.03–1.92, *p* = 0.030). The pathological N stage was also independently associated with DFS (HR = 1.30, 95% CI 1.00–1.68, *p* = 0.049) but not with OS (HR = 1.15, 95% CI 0.88–1.51, *p* = 0.300). Notably, after accounting for other prognostic factors, the duration of the 5-FU infusion was not identified as an independent prognostic factor for either DFS (HR = 1.03, 95% CI 0.51–2.07, *p* = 0.940) or OS (HR = 0.87, 95% CI 0.43–1.77, *p* = 0.707). The clinical T stage, clinical N stage, age, and sex did not reach significance final as independent prognostic factors in the final model (*p* > 0.05).

## 4. Discussion

This study presents a real-world data analysis evaluating the effects of different infusion durations on the clinical response, pathological response, toxicity profile, and survival outcomes in gastric and gastroesophageal junction adenocarcinomas. Our findings show that a 48 h infusion duration offers similar efficacy but a more tolerable toxicity profile than a 24 h regimen. These results support the view that the infusion duration of fluorouracil is an important variable that affects treatment tolerability from both pharmacokinetic and pharmacodynamic perspectives. In particular, the 48 h infusion protocol reduced non-hematological toxicities (such as loss of appetite and fatigue) and decreased dose-delay rates. This finding is clinically significant because of its potential to increase treatment continuity and patient compliance. The results obtained were consistent with those of previous studies on fluorouracil-based chemotherapy protocols. Several studies have reported that long-term, low-intensity infusions offer a more balanced pharmacokinetic profile, reducing fluctuations in the plasma concentration and thus minimizing gastrointestinal and hematological toxicities. Moreover, prolonging the infusion duration does not reduce antitumor efficacy; in contrast, in certain subgroups, increased tolerance leads to higher treatment completion rates. Recent studies have demonstrated that both 24 h and 48 h infusion regimens exhibit similar efficacy and side effect profiles, suggesting that patients can have some flexibility in their treatment schedules [17,18]. Reports from clinical studies have highlighted that side effects are generally mild and that nausea is observed in a small percentage of cases. The choice between infusion durations should be individualized and made according to patient needs and preferences, considering factors such as treatment tolerance and potential side effects. The neoadjuvant FLOT protocol became standard following the FLOT4-AIO trial [5]. Notably, the recent MATTERHORN phase 3 trial demonstrated that the addition of perioperative durvalumab to FLOT significantly improves event-free survival compared with FLOT alone in patients with resectable gastric or GEJ adenocarcinoma, further advancing the therapeutic landscape [6]. Although 5-FU was administered as a 24 h infusion in the original FLOT4-AIO study, some clinical practice centers have reported the use of 46–48 h home-based pump applications with similar efficacy. In our analysis, the lack of a difference between the groups in terms of radiological (*p* = 0.922) and pathological (*p* = 0.362) response supports the idea that extending the duration of 5-FU infusions in the FLOT protocol does not reduce treatment efficacy. The lack of significant differences in OS and PFS in survival analyses may be explained by the maintenance of the fluorouracil dose intensity. Studies demonstrating an association between dose intensity and survival support this finding [19,20]. The 12–13% pCR rate observed in both groups is consistent with the FLOT4-AIO study (15.6% in the FLOT arm) and other real-world studies, confirming the efficacy of both infusion schedules [5,21]. While the original FLOT4 protocol utilized a 24 h infusion method, many centers worldwide have adopted the 48 h infusion regimen, based on data from other fluoropyrimidine protocols and theoretical pharmacokinetic advantages [9,10]. Several factors may explain the comparable efficacy of the two infusion durations. First, the total 5-FU dose and dose intensity were the same between the groups, with only the administration schedule differing. Second, 5-FU rapidly reaches stable plasma concentrations, and its cytotoxic effects may plateau after the first 24 h of continuous infusion. Third, the synergistic effects of docetaxel and oxaliplatin in the FLOT regimen may overshadow the minor differences in 5-FU pharmacodynamics. When examining histopathological characteristics, the higher rate of signet ring cell carcinoma in the 24 h group suggests more aggressive biological behavior [22]. However, despite this biological disadvantage, the similarity in survival and treatment response outcomes supports the idea that the infusion duration is independent of tumor biology. Our multivariate analysis identified ypT and ypN stages as the most significant independent prognostic factors for survival—consistent with the extensive literature showing that the pathological response to neoadjuvant chemotherapy is the strongest determinant of long-term outcomes in gastric cancer [23,24]. In particular, despite a higher initial cT stage in the 24 h group, similar prognostic outcomes were observed when postoperative pathological staging was considered. Effective tumor downstaging was achieved using both infusion programs. Toxicity profiles were comparable across the groups, with no significant differences in grade 3–4 adverse events. This finding is especially important considering the concerns that shorter infusion durations could increase drug concentrations and related toxicities. The slightly higher rate of dose delays in the 24 h group (35.7% vs. 12.9%), despite similar overall grade 3–4 toxicity rates, may reflect the higher initial tumor burden (cT stage) rather than infusion-related toxicity. Differences in tumor localization may also affect clinical outcomes. Notably, differences in the biology and treatment response between GEJ and distal gastric tumors have been previously identified. However, our study demonstrated that the infusion duration yielded similar response and survival outcomes regardless of tumor localization, suggesting that it is not a prognostic factor. Nevertheless, both regimens had advantages. Practically speaking, with 24 h infusions, each cycle reduces hospital stays by 24 h, potentially lowering healthcare costs and infection risk [25] while improving patient comfort and quality of life. In settings with limited resources or during times of high bed occupancy, a shorter infusion duration can improve access to treatment without compromising the outcome. Additionally, reducing the time that patients are connected to infusion pumps may decrease the risk of catheter-related complications. In contrast, in our study, the rate of dose delays was significantly lower with 48 h infusions (*p* = 0.012). Previous large-cohort analyses have shown that dose delays and dose reductions negatively affect survival. In this context, the potential for 48 h infusions to increase treatment continuity via improved tolerability is a clinically significant finding. The limitations of our study include its retrospective design and limited number of patients. Nevertheless, this analysis, which compares the two commonly used infusion durations in clinical practice, provides important guidance for treatment planning.

Several additional study limitations warrant acknowledgment in the context of the discussion above. Although propensity score matching was employed to mitigate baseline imbalances, residual confounding persisted, particularly for the clinical T stage (*p* = 0.009 in the matched cohort vs. *p* < 0.001 in the original cohort). Although matching substantially improved the overall balance (average standardized mean difference reduced from 0.317 to 0.211), this persistent imbalance likely reflects unmeasured factors influencing the institutional choice of infusion duration that could not be fully balanced through matching alone. Because PSM can only adjust for measured confounders, unmeasured variables such as ECOG performance status, comorbidities, physician preference, and referral patterns may have contributed to residual bias and could partly explain the observed differences in dose delays and tolerability between groups. Tighter matching with a caliper (0.2 SD) further improved the balance but resulted in considerable patient loss (28→13 patients in the 24 h group), reducing statistical power and illustrating the inherent trade-off between balance and sample size in observational studies. Together with the retrospective design and limited cohort size, these factors restrict the strength of causal inferences and reinforce the hypothesis-generating nature of our findings, supporting the need for prospective validation.

## 5. Conclusions

In this retrospective multicenter study, 24 h and 48 h 5-FU infusion durations within the perioperative FLOT regimen demonstrated comparable pathological complete response rates (10.7% vs. 12.9%), disease-free survival, overall survival, and toxicity profiles in patients with gastric or gastroesophageal junction adenocarcinoma. These findings were consistent both in the original cohort and after propensity score matching and were further confirmed by a multivariate Cox regression analysis, which identified pathological T and N stages—but not infusion duration—as independent prognostic factors for survival. The 48 h infusion schedule was associated with significantly fewer dose delays (12.9% vs. 35.7%, *p* = 0.012), suggesting a tolerability advantage that may enhance treatment continuity and patient compliance in clinical practice. Conversely, the 24 h schedule offers practical benefits, including shorter hospitalization durations per cycle and potential reductions in healthcare resource utilization. These results suggest that both infusion schedules are clinically acceptable and that the choice between them may reasonably be individualized based on patient preferences, institutional resources, and tolerability considerations, without compromising oncological outcomes. However, the inherent limitations of the retrospective design, small sample size, residual confounding despite propensity score matching, and severely limited statistical power (~10% for the primary endpoint) preclude definitive conclusions. These hypothesis-generating findings underscore the need for prospective, randomized controlled trials to definitively establish the optimal 5-FU infusion duration within the perioperative FLOT framework for gastric and gastroesophageal junction adenocarcinoma.

## Figures and Tables

**Table 1:** Baseline patient and tumor characteristics.

Characteristic	24 h (*n* = 30 Matched)	48 h (*n* = 60 Matched)	*p*
Demographics			
Age (years), mean ± SD	61.7 ± 9.2	59.8 ± 10.6	0.393
Male gender, *n* (%)	22 (78.6)	53 (62.4)	0.166
Tumor Characteristics			
Histology: adenocarcinoma, *n* (%)	25 (89.3)	75 (88.2)	
cT stage, median (IQR) *	**3 (3–4)**	**3 (2–3)**	**<0.001**
cN stage, median (IQR)	2 (1–2)	2 (1–2)	
Treatment Parameters			
Neoadjuvant cycles, mean ± SD	4.2 ± 0.6	4.1 ± 0.7	
**Dose delay,** ***n*** (%) *	**10 (35.7)**	**11 (12.9)**	**0.012**
Surgery performed, *n* (%)	26 (92.9)	82 (96.5)	
Grade 3–4 Toxicity			
Neutropenia, *n* (%)	3 (11.1)	4 (4.8)	
Anemia, *n* (%)	1 (3.7)	2 (2.4)	
Thrombocytopenia, *n* (%)	1 (3.7)	1 (1.2)	
Nausea/vomiting, *n* (%)	2 (7.4)	1 (1.2)	
Fatigue, *n* (%)	1 (3.7)	5 (6.0)	
Any grade 3–4 toxicity, *n* (%)	7 (25.0)	17 (20.0)	

SD, standard deviation; IQR, interquartile range; cT, clinical tumor stage; cN, clinical nodal stage. * *p* < 0.05 (bold values indicate statistical significance). All other comparisons *p* > 0.05.

**Table 3:** Survival outcomes.

Survival Metric	24 h (*n* = 28)	48 h (*n* = 85)	Log-Rank *p*-Value
Disease-Free Survival			
Median DFS (months)	27.4	NR	0.887
1-year DFS (%)	74.4	72.3	
2-year DFS (%)	65.1	61.6	
Overall Survival			
Median OS (months)	32.8	NR	0.70
1-year OS (%)	88.4	85.5	
2-year OS (%)	75.7	65.7	

DFS, disease-free survival; OS, overall survival; NR, not reached. No significant differences between groups (log-rank test *p* > 0.05).

**Table 4:** Multivariate Cox proportional hazard regression analysis.

Variable	DFS HR	DFS 95% CI	*p*	OS HR	OS 95% CI	*p*
5-FU infusion 24 h vs. 48 h	1.03	0.51–2.07	0.940	0.87	0.43–1.77	0.707
Age (per year)	1.00	0.97–1.03	0.895	1.02	0.99–1.05	0.248
Male gender	1.58	0.86–2.90	0.137	1.65	0.88–3.07	0.117
cT stage (per stage)	0.86	0.55–1.34	0.508	0.98	0.62–1.54	0.931
cN stage (per stage)	1.41	0.99–2.00	0.055	1.26	0.90–1.78	0.182
pCR achievement	1.02	0.29–3.51	0.978	0.94	0.27–3.21	0.919
ypT stage (per stage) *	**1.45**	**1.07–1.95**	**0.015**	**1.41**	**1.03–1.92**	**0.030**
ypN stage (per stage) *	**1.30**	**1.00–1.68**	**0.049**	1.15	0.88–1.51	0.300

HR, hazard ratio; CI, confidence interval; DFS, disease-free survival; OS, overall survival; pCR, pathologic complete response; cT/cN, clinical tumor/nodal stage; ypT/ypN, pathologic tumor/nodal stage after neoadjuvant therapy. * *p* < 0.05 (bold values indicate independent prognostic significance).

## Data Availability

The raw data supporting the conclusions of this article will be made available by the authors on request.

## References

[1] Morgan E., Arnold M., Camargo M.C., Gini A., Kunzmann A.T., Matsuda T., Meheus F., Verhoeven R.H., Vignat J., Laversanne M. (2022). The current and future incidence and mortality of gastric cancer in 185 countries, 2020–2040: A population-based modelling study. eClinicalMedicine.

[2] Ajani J.A., D’Amico T.A., Almhanna K., Bentrem D.J., Chao J., Das P., Denlinger C.S., Fanta P., Farjah F., Fuchs C.S. (2016). Gastric Cancer, Version 3.2016, NCCN Clinical Practice Guidelines in Oncology. J. Natl. Compr. Canc. Netw..

[3] Sundar R., Nakayama I., Markar S.R., Shitara K., van Laarhoven H.W.M., Janjigian Y.Y., Smyth E.C. (2025). Gastric cancer. Lancet.

[4] Lordick F., Carneiro F., Cascinu S., Fleitas T., Haustermans K., Piessen G., Vogel A., Smyth E. (2022). Gastric cancer: ESMO Clinical Practice Guideline for diagnosis, treatment and follow-up. Ann. Oncol..

[5] Al-Batran S.E., Homann N., Pauligk C., Goetze T.O., Meiler J., Kasper S., Kopp H.-G., Mayer F., Haag G.M., Luley K. (2019). Perioperative chemotherapy with fluorouracil plus leucovorin, oxaliplatin, and docetaxel versus fluorouracil or capecitabine plus cisplatin and epirubicin for locally advanced, resectable gastric or gastro-oesophageal junction adenocarcinoma (FLOT4): A randomised, phase 2/3 trial. Lancet.

[6] Janjigian Y.Y., Al-Batran S.E., Wainberg Z.A., Muro K., Molena D., Van Cutsem E., Hyung W.J., Wyrwicz L., Oh D.Y., Omori T. (2025). Perioperative Durvalumab in Gastric and Gastroesophageal Junction Cancer. N. Engl. J. Med..

[7] Dos Santos M., Lequesne J., Leconte A., Corbinais S., Parzy A., Guilloit J.-M., Varatharajah S., Brachet P.-E., Dorbeau M., Vaur D. (2022). Perioperative treatment in resectable gastric cancer with spartalizumab in combination with fluorouracil, leucovorin, oxaliplatin and docetaxel (FLOT): A phase II study (GASPAR). BMC Cancer.

[8] ClinicalTrials.gov A Study of Total Neoadjuvant Chemotherapy with FLOT VS Standard Perioperative FLOT in Patients with Gastric or GEJ Cancer (NCT05567835). NCT05567835.

[9] Piedbois P., Rougier P., Buyse M., Pignon J., Ryan L., Hansen R., Zee B., Weinerman B., Pater J., Meta-Analysis Group in Cancer (1998). Efficacy of intravenous continuous infusion of fluorouracil compared with bolus administration in advanced colorectal cancer. J. Clin. Oncol..

[10] Grem J.L., Quinn M., Ismail A.S., Takimoto C.H., Lush R., Liewehr D.J., Steinberg S.M., Balis F.M., Chen A.P., Monahan B.P. (2001). Pharmacokinetics and pharmacodynamic effects of 5-fluorouracil given as a one-hour intravenous infusion. Cancer Chemother. Pharmacol..

[11] Lordick F., Lorenzen S., Stollfuss J., Vehling-Kaiser U., Kullmann F., Hentrich M., Zumschlinge R., Dietzfelbinger H., Thoedtmann J., Hennig M. (2005). Phase II study of weekly oxaliplatin plus infusional fluorouracil and folinic acid (FUFOX regimen) as first-line treatment in metastatic gastric cancer. Br. J. Cancer.

[12] Möhring C., Mańczak A., Timotheou A., Sadeghlar F., Zhou T., Mahn R., Monin M.B., Toma M., Feldmann G., Brossart P. (2023). Perioperative therapy with FLOT4 significantly increases survival in patients with gastroesophageal and gastric cancer in a large real-world cohort. Int. J. Cancer.

[13] Bhargava P., Das S., Ostwal V., Srinivas S., Bhandare M., Chaudhari V., Bal M., Mantri A., Kapoor A., Shrikhande S.V. (2022). An Analysis of Tolerance and Early Survival Outcomes with Perioperative Modified FLOT in Gastric Cancers. South Asian J. Cancer.

[14] Dawood T., Rashid Y.A., Khan S.R., Jabbar A.A., Zahir M.N., Moosajee M.S. (2024). Outcomes of locally advanced gastric and gastroesophageal adenocarcinoma cancers treated with neoadjuvant FLOT in a tertiary care hospital in Pakistan. Ecancermedicalscience.

[15] Villanueva L., Anabalon J., Butte J.M., Salman P., Panay S., Milla E., Gallardo C., Hoefler S., Charles R., Reyes F. (2021). Total neoadjuvant chemotherapy with FLOT scheme in resectable adenocarcinoma of the gastro-oesophageal junction or gastric adenocarcinoma: Impact on pathological complete response and safety. Ecancermedicalscience.

[16] Cancer Research UK Fluorouracil, Leucovorin, Oxaliplatin and Docetaxel (FLOT). https://www.cancerresearchuk.org/about-cancer/treatment/drugs/fluorouracil-leucovorin-oxaliplatin-docetaxel-flot.

[17] Tastekin D., Paksoy N., Dogan I., Ferhatoglu F., Khanmammadov N., Bozbey H.U., Karabulut S. (2023). Fluorouracil, leucovorin, oxaliplatin, and docetaxel (FLOT) regimen in the first-line treatment of metastatic gastric cancer: A single-center experience. J. Cancer Res. Ther..

[18] Heckl S.M., Behrens H.M., Ebert U., Ulase D., Richter F., Becker T., Letsch A., Röcken C. (2025). Impact of adjuvant therapy on outcomes of cancer of the stomach and gastroesophageal junction in the real-world. Gastric Cancer.

[19] Steventon L., Man K.K.C., Nicum S., Miller R., Hasson S.P., Shah S., Baser M., Kipps E., Forster M.D., Almossawi O. (2024). The impact of inter-cycle treatment delays on overall survival in patients with advanced-stage ovarian cancer. Oncologist.

[20] Nielson C.M., Bylsma L.C., Fryzek J.P., Saad H.A., Crawford J. (2021). Relative Dose Intensity of Chemotherapy and Survival in Patients with Advanced Stage Solid Tumor Cancer: A Systematic Review and Meta-Analysis. Oncologist.

[21] Homann N., Pauligk C., Luley K., Kraus T.W., Bruch H., Atmaca A., Noack F., Altmannsberger H., Jäger E., Al-Batran S. (2012). Pathological complete remission in patients with oesophagogastric cancer receiving preoperative 5-fluorouracil, oxaliplatin and docetaxel. Int. J. Cancer.

[22] del Arco C.D., Aceñero M.J.F., Medina L.O. (2024). Molecular Classifications in Gastric Cancer: A Call for Interdisciplinary Collaboration. Int. J. Mol. Sci..

[23] Becker K., Mueller J.D., Schulmacher C., Ott K., Fink U., Busch R., Bottcher K., Siewert J.R., Hofler H. (2003). Histomorphology and grading of regression in gastric carcinoma treated with neoadjuvant chemotherapy. Cancer.

[24] Mansour J.C., Tang L., Shah M., Bentrem D., Klimstra D.S., Gonen M., Kelsen D.P., Brennan M.F., Coit D.G. (2007). Does graded histologic response after neoadjuvant chemotherapy predict survival for completely resected gastric cancer?. Ann. Surg. Oncol..

[25] Wagner A.D., Grothe W., Haerting J., Kleber G., Grothey A., Fleig W.E. (2006). Chemotherapy in advanced gastric cancer: A systematic review and meta-analysis based on aggregate data. J. Clin. Oncol..

